# Hermitian-Randić matrix and Hermitian-Randić energy of mixed graphs

**DOI:** 10.1186/s13660-017-1329-8

**Published:** 2017-03-03

**Authors:** Yong Lu, Ligong Wang, Qiannan Zhou

**Affiliations:** Department of Applied Mathematics, School of Science, Northwestern Polytechnical University, Xi’an, Shaanxi 710072 People’s Republic of China

**Keywords:** 05C50, 05C07, 05C31, mixed graph, Hermitian-adjacency matrix, Hermitian-Randić matrix, Hermitian-Randić energy

## Abstract

Let *M* be a mixed graph and $H(M)$ be its Hermitian-adjacency matrix. If we add a Randić weight to every edge and arc in *M*, then we can get a new weighted Hermitian-adjacency matrix. What are the properties of this new matrix? Motivated by this, we define the Hermitian-Randić matrix $R_{H}(M)=(r_{h})_{kl}$ of a mixed graph *M*, where $(r_{h})_{kl}=-(r_{h})_{lk}=\frac{\mathbf{i}}{\sqrt {d_{k}d_{l}}}$ ($\mathbf{i}=\sqrt{-1}$) if $(v_{k},v_{l})$ is an arc of *M*, $(r_{h})_{kl}=(r_{h})_{lk}=\frac{1}{\sqrt{d_{k}d_{l}}}$ if $v_{k}v_{l}$ is an undirected edge of *M*, and $(r_{h})_{kl}=0$ otherwise. In this paper, firstly, we compute the characteristic polynomial of the Hermitian-Randić matrix of a mixed graph. Furthermore, we give bounds on the Hermitian-Randić energy of a general mixed graph. Finally, we give some results about the Hermitian-Randić energy of mixed trees.

## Introduction

In this paper, we only consider simple graphs without multiedges and loops. A graph *M* is said to be *mixed* if it is obtained from an undirected graph $M_{U}$ by orienting a subset of its edges. We call $M_{U}$ the *underlying graph* of *M*. Clearly, a mixed graph concludes both possibilities of all edges oriented and all edges undirected as extreme cases.

Let *M* be a mixed graph with vertex set $V(M)=\{v_{1},v_{2},\ldots ,v_{n}\}$ and edge set $E(M)$. For $v_{i},v_{j}\in V(M)$, we denote an undirected edge joining two vertices $v_{i}$ and $v_{j}$ of *M* by $v_{i}v_{j}$ (or $v_{i}\leftrightarrow v_{j}$). Denote a directed edge (or arc) from $v_{i}$ to $v_{j}$ by $(v_{i},v_{j})$ (or $v_{i}\rightarrow v_{j}$). In addition, let $E_{0}(M)$ denote the set of all undirected edges and $E_{1}(M)$ denote all the directed arcs set. Clearly, $E(M)$ is the union of $E_{0}(M)$ and $E_{1}(M)$. A mixed graph is called *mixed tree* (or *mixed bipartite graph*) if its underlying graph is a tree (or bipartite graph). In general, the order, size, number of components and degree of a vertex of *M* are the same to those in $M_{U}$. We use Bondy and Murty [1] for terminologies and notations not defined here.

Let *G* be a simple graph with vertex set $\{v_{1},v_{2},\ldots,v_{n}\} $. The *adjacency matrix* of a simple graph *G* of order *n* is defined as the $n\times n$ symmetric square matrix $A=A(G)=(a_{ij})$, where $a_{ij}=1$ if $v_{i}v_{j}$ is an edge of *G*, otherwise $a_{ij}=0$. We denote by $d_{i}=d(v_{i})=d_{G}(v_{i})$ ($i=1,2,\ldots,n$) the degree of vertex $v_{i}$. In addition, for a mixed graph *M*, if $v_{i}\in V(M)$, then we also denote $d_{i}=d(v_{i})=d_{M_{U}}(v_{i})$. The *energy* of the graph *G* (see the survey of Gutman, Li and Zhang [2] and the book of Li, Shi and Gutman [3]) is defined as $\mathcal{E}_{A}(G)=\sum_{i=1}^{n}|\rho_{i}|$, where $\rho_{1},\rho _{2},\ldots,\rho_{n}$ are all eigenvalues of $A(G)$.

A convenient parameter of *G* is the *general Randić index* $R_{\alpha}(G)$ defined as $R_{\alpha}(G)=\sum_{uv\in E(G)}(d_{u}d_{v})^{\alpha}$, where the summation is over all (unordered) edges *uv* in *G*. The molecular structure-descriptor, first proposed by Randić [4] in 1975, is defined as the sum of $\frac{1}{\sqrt{d_{u}d_{v}}}$ over all edges *uv* of *G* (with $\alpha=-\frac{1}{2}$). Nowadays, $R=R(G)=\sum_{uv\in E(G)}\frac{1}{\sqrt{d_{u}d_{v}}}$ of *G* is referred to as the *Randić index*. Countless chemical applications, the mathematical properties and mathematical chemistry of the Randić index were reported in [5–7].

Gutman et al. [8] pointed out that the Randić-index-concept is purposeful to associate the graph *G* with a symmetric square matrix of order *n*, named *Randić matrix* $R(G)=(r_{ij})$, where $r_{ij}=\frac{1}{\sqrt{d_{i}d_{j}}}$ if $v_{i}v_{j}$ is an edge of *G*, otherwise $r_{ij}=0$. Let $D(G)$ be the diagonal matrix of vertex degrees of *G*. If *G* has no isolated vertices, then $R(G)=D(G)^{-\frac {1}{2}}A(G)D(G)^{-\frac{1}{2}}$.

The concept of *Randić energy* of a graph *G*, denoted by $\mathcal{E}_{R}(G)$, was introduced in [9] as $\mathcal {E}_{R}(G)=\sum_{i=1}^{n}|\gamma_{i}|$, where $\gamma_{i}$ is the eigenvalues of $R(G)$, $i=1,2,\ldots,n$. Some basic properties of the Randić index, Randić matrix and Randić energy were determined in the papers [8–20].

An oriented graph $G^{\sigma}$ is a digraph which assigns each edge of *G* a direction *σ*. The *skew adjacency matrix* associated to $G^{\sigma}$ is the $n\times n$ matrix $S(G^{\sigma})=(s_{ij})$, where $s_{ij}=-s_{ji}=1$ if $(v_{i},v_{j})$ is an arc of $G^{\sigma}$, otherwise $s_{ij}=s_{ji}=0$. The *skew energy* of $G^{\sigma}$, denoted by $\mathcal{E}_{S}(G^{\sigma})$, is defined as the sum of the norms of all the eigenvalues of $S(G^{\sigma})$. For more details about skew energy, we can refer to [21, 22].

In 2016, Gu, Huang and Li [14] defined the *skew Randić matrix* $R_{s}(G^{\sigma})=((r_{s})_{ij})$ of an oriented graph $G^{\sigma}$ of order *n*, where $(r_{s})_{ij}=-(r_{s})_{ji}=\frac {1}{\sqrt{d_{i}d_{j}}}$ if $(v_{i},v_{j})$ is an arc of $G^{\sigma}$, otherwise $(r_{s})_{ij}=(r_{s})_{ji}=0$. Let $D(G)$ be the diagonal matrix of vertex degrees of *G*. If $G^{\sigma}$ has no isolated vertices, then $R_{s}(G^{\sigma})=D(G)^{-\frac{1}{2}}S(G^{\sigma })D(G)^{-\frac{1}{2}}$.

The *Hermitian-adjacency matrix* of a mixed graph *M* of order *n* is the $n\times n$ matrix $H(M)=(h_{kl})$, where $h_{kl}=-h_{lk}= \mathbf{i}$ ($\mathbf{i}=\sqrt{-1}$) if $(v_{k},v_{l})$ is an arc of *M*, $h_{kl}=h_{lk}=1$ if $v_{k}v_{l}$ is an undirected edge of *M*, and $h_{kl}=0$ otherwise. Obviously, $H(M)=H(M)^{\ast}:={\overline{H(M)}}^{T}$. Thus all its eigenvalues are real. This matrix was introduced by Liu and Li in [23] and independently by Guo and Mohar in [24]. The *Hermitian energy* of a mixed graph *M* is defined as $\mathcal {E}_{H}(M)=\sum^{n}_{i=1}|\lambda_{i}|$, where $\lambda_{1},\lambda _{2},\ldots,\lambda_{n}$ are all eigenvalues of $H(M)$. Denote by $\operatorname{Sp}_{H}(M)=(\lambda_{1},\lambda_{2},\ldots,\lambda_{n})$ the *spectrum* of $H(M)$. For more details about the Hermitian-adjacency matrix and the Hermitian energy of mixed graphs, we can refer to [23–28].

From the above we can see that if we add a Randić weight to every edge in a simple graph *G*, then we can get a Randić matrix $R(G)$. If we add a Randić weight to every arc in an oriented graph $G^{\sigma}$, then we can get a skew Randić matrix $R_{s}(G^{\sigma})$. Let *M* be a mixed graph and $H(M)$ be its Hermitian-adjacency matrix. If we add a Randić weight to every edge and arc in *M*, then we can get a new weighted Hermitian-adjacency matrix. What are the properties of this new matrix? Motivated by this, we define the Hermitian-Randić matrix of a mixed graph *M*.

Let *M* be a mixed graph on the vertex set $\{v_{1},v_{2},\ldots,v_{n}\} $, then the *Hermitian-Randić matrix* of *M* is the $n\times n$ matrix $R_{H}(M)=((r_{h})_{kl})$, where

$$(r_{h})_{kl}=\left \{\ \textstyle\begin{array}{l@{\quad}l} \frac{1}{\sqrt{d_{k}d_{l}}},&\text{if } v_{k}\leftrightarrow v_{l},\\ \frac{\mathbf{i}}{\sqrt{d_{k}d_{l}}},&\text{if } v_{k}\rightarrow v_{l},\\ \frac{-\mathbf{i}}{\sqrt{d_{k}d_{l}}},&\text{if } v_{l}\rightarrow v_{k},\\ 0,&\text{otherwise}. \end{array}\displaystyle \right . $$

Let $D(M_{U})$ be the diagonal matrix of vertex degrees of $M_{U}$. If *M* has no isolated vertices, then $R_{H}(M)=D(M_{U})^{-\frac {1}{2}}H(M)D(M_{U})^{-\frac{1}{2}}$. For a mixed graph *M*, let $R_{H}(M)$ be its Hermitian-Randić matrix. It is obvious that $R_{H}(M)$ is a Hermitian matrix, so all its eigenvalues $\mu_{1},\mu_{2},\ldots,\mu_{n}$ are real. The *spectrum* of $R_{H}(M)$ is defined as $\operatorname{Sp}_{R_{H}}(M)=(\mu_{1},\mu _{2},\ldots,\mu_{n})$. The energy of $R_{H}(M)$, denoted by $\mathcal {E}_{R_{H}}(M)$, is called *Hermitian-Randić energy*, which is defined as the sum of the absolute values of its eigenvalues of $R_{H}(M)$, that is, $\mathcal{E}_{R_{H}}(M)=\sum^{n}_{i=1}|\mu_{i}|$.

In this paper, we define the Hermitian-Randić matrix of a mixed graph *M* and study some basic characteristics of the Hermitian-Randić matrix of mixed graphs. In Section 2, we give the characteristic polynomial of the Hermitian-Randić matrix of a mixed graph *M*. In Section 3, we study some bounds on the Hermitian-Randić energy of mixed graphs with different parameters and give the conditions under which mixed graphs can attain those Hermitian-Randić energy bounds. In Section 4, we show that the Hermitian-Randić energy of a mixed tree is the same as the Randić energy of its underlying graph. In Section 5, we summarize the results of this paper and give some future works we will study.

## Hermitian-Randić characteristic polynomial of a mixed graph

In this section, we will give the characteristic polynomial of a Hermitian-Randić matrix of a mixed graph *M*, i.e., the $R_{H}$-characteristic polynomial of *M*. At first, we will introduce some basic definitions.

The *value* of a mixed walk $W=v_{1}v_{2}\cdots v_{l}$ is $r_{h}(W)=(r_{h})_{12}(r_{h})_{23}(r_{h})_{(l-1)l}$. A mixed walk *W* is *positive* (or *negative*) if $r_{h}(W)=\frac{1}{\sqrt {d_{1}d_{l}}d_{2}d_{3}\cdots d_{(l-1)}}$ (or $r_{h}(W)=-\frac{1}{\sqrt {d_{1}d_{l}}d_{2}d_{3}\cdots d_{(l-1)}}$). Note that for one direction the value of a mixed walk or a mixed cycle is *α*, then for the reversed direction its value is *α̅*. Thus, if the value of a mixed cycle *C* is $\prod_{v_{j}\in V(C)}\frac{1}{d(v_{j})}$ (resp. $-\prod_{v_{j}\in V(C)}\frac{1}{d(v_{j})}$) in a direction, then its value is $\prod_{v_{j}\in V(C)}\frac{1}{d(v_{j})}$ (resp. $-\prod_{v_{j}\in V(C)}\frac{1}{d(v_{j})}$) for the reversed direction. In these situations, we just term this mixed cycle a positive (resp. negative) mixed cycle without mentioning any direction.

If each mixed cycle is positive (resp. negative) in a mixed graph *M*, then *M* is *positive* (resp. *negative*). A mixed graph *M* is called an *elementary graph* if every component of *M* is an edge, an arc or a mixed cycle, where every edge-component in *M* is defined to be positive. A *real spanning elementary subgraph* of a mixed graph *M* is an elementary subgraph such that it contains all vertices of *M* and all its mixed cycles are real.

Now we will give two results which are similar to those in [23, 29, 30].

Let *M* be a mixed graph of order *n* with its Hermitian-Randić matrix $R_{H}(M)$. Denote the $R_{H}$ *-characteristic polynomial* of $R_{H}(M)$ of *M* by

$$P_{R_{H}}(M,x)=\operatorname {det}\bigl(xI-R_{H}(M) \bigr)=x^{n}+a_{1}x^{n-1}+a_{2}x^{n-2}+ \cdots+a_{n}. $$

### Theorem 2.1

*Let* $R_{H}(M)$ *be the Hermitian*-*Randić matrix of a mixed graph* *M* *of order* *n*. *Then*

$$\operatorname{det}R_{H}(M)=\sum_{M'}(-1)^{r(M')+l(M')}2^{s(M')}W \bigl(M'\bigr), $$

*where the summation is over all real spanning elementary subgraphs* $M'$ *of* *M*, $r(M')=n-c(M')$, $c(M')$ *denotes the number of components of* $M'$, $l(M')$ *denotes the number of negative mixed cycles of* $M'$, $s(M')$ *denotes the number of mixed cycles with length* ≥3 *in* $M'$, $W(M')=\prod_{v_{i}\in V(M')}\frac{1}{d_{M_{U}}(v_{i})}$.

### Proof

Let *M* be a mixed graph of order *n* with vertex set $\{ v_{1},v_{2},\ldots, v_{n}\}$. Then

$$\operatorname{det}R_{H}(M)=\sum_{\pi\in S_{n}}\operatorname{sgn}(\pi) (r_{h})_{1\pi (1)}(r_{h})_{2\pi(2)} \cdots(r_{h})_{n\pi(n)}, $$

where $S_{n}$ is the set of all permutations on $\{1,2,\ldots,n\}$.

Consider a term $\operatorname{sgn}(\pi)(r_{h})_{1\pi(1)}(r_{h})_{2\pi(2)}\cdots (r_{h})_{n\pi(n)}$ in the expansion of $\operatorname{det}R_{H}(M)$. If $v_{k}v_{\pi(k)}$ is not an edge or arc of *M*, then $(r_{h})_{k\pi (k)}=0$; that is, this term vanishes. Thus, if the term corresponding to a permutation *π* is non-zero, then *π* is fixed-point-free and can be expressed uniquely as the composition of disjoint cycles of length at least 2. Consequently, each non-vanishing term in the expansion of $\operatorname{det}R_{H}(M)$ gives rise to an elementary mixed graph $M'$ of *M* with $V(M')=V(M)$. That is, $M'$ is a spanning elementary subgraph of *M* of order *n*.

A spanning elementary subgraph $M'$ of *M* with $s(M')$ number of mixed cycles (length ≥3) gives $2^{s(M')}$ permutations *π* since, for each mixed cycle-component in $M'$, there are two ways of choosing the corresponding cycles in *π*. For a vertex $v_{k}\in V(M')$, we denote $d_{k}=d(v_{k})=d_{M_{U}}(v_{k})$. Furthermore, if for some direction of a permutation *π*, a mixed cycle-component $C_{1}$ has value $\mathbf{i}\prod_{v_{j}\in V(C_{1})}\frac{1}{d(v_{j})}$ (or $-\mathbf{i}\prod_{v_{j}\in V(C_{1})}\frac{1}{d(v_{j})}$), then for the other direction $C_{1}$ has value $-\mathbf {i}\prod_{v_{j}\in V(C_{1})}\frac{1}{d(v_{j})}$ (or $\mathbf {i}\prod_{v_{j}\in V(C_{1})}\frac{1}{d(v_{j})}$) and vice versa. Thus, they cancel each other in the summation. In addition, if for some direction of a permutation *π*, $C_{1}$ has value $\prod_{v_{j}\in V(C_{1})}\frac{1}{d(v_{j})}$ (or $-\prod_{v_{j}\in V(C_{1})}\frac {1}{d(v_{j})}$), then for the other direction $C_{1}$ has the same value. For each edge-component $(kl)$ corresponding to the factors $(r_{h})_{kl}(r_{h})_{lk}$ has value $\frac{1}{\sqrt{d_{k}d_{l}}}\frac {1}{\sqrt{d_{l}d_{k}}}=\frac{1}{d_{k}d_{l}}$. For each arc-component $(kl)$ corresponding to the factors $(r_{h})_{kl}(r_{h})_{lk}$ has value $\frac{\mathbf{i}\cdot(-\mathbf{i})}{\sqrt {d_{k}d_{l}}\sqrt{d_{l}d_{k}}}=\frac{1}{d_{k}d_{l}}$.

Since $\operatorname{sgn}(\pi)=(-1)^{n-c(M')}=(-1)^{r(M')}$ and each real spanning elementary subgraph $M'$ contributes $(-1)^{r(M')+l(M')}2^{s(M')}\prod_{v_{i}\in V(M')}\frac{1}{d_{M_{U}}(v_{i})}$ to the determinant of $R_{H}(M)$. This completes the proof. □

Now, we shall obtain a description of all the coefficients of the characteristic polynomial $P_{R_{H}}(M,x)$ of a mixed graph *M*.

### Theorem 2.2

*For a mixed graph* *M*, *if the* $R_{H}$-*characteristic polynomial of* *M* *is denoted by* $P_{R_{H}}(M,x)=\operatorname {det}(xI-R_{H}(M))=x^{n}+a_{1}x^{n-1}+a_{2}x^{n-2}+\cdots+a_{n}$, *then the coefficients of* $P_{R_{H}}(M,x)$ *are given by*

$$(-1)^{k}a_{k}=\sum_{M'}(-1)^{r(M')+l(M')}2^{s(M')} \prod_{v_{i}\in V(M')}\frac{1}{d_{M_{U}}(v_{i})}, $$

*where the summation is over all real elementary subgraphs* $M'$ *with order* *k* *of* *M*, $r(M')=k-c(M')$, $c(M')$ *denotes the number of components of* $M'$, $l(M')$ *denotes the number of negative mixed cycles of* $M'$, $s(M')$ *denotes the number of mixed cycles with length* ≥3 *in* $M'$.

### Proof

The proof follows from Theorem 2.1 and the fact that $(-1)^{k}a_{k}$ is the summation of determinants of all principal $k\times k$ submatrices of $R_{H}(M)$. □

### Corollary 2.3

*For a mixed graph* *M*, *let the* $R_{H}$-*characteristic polynomial of* *M* *be denoted by* $P_{R_{H}}(M,x)=\operatorname {det}(xI-R_{H}(M))=x^{n}+a_{1}x^{n-1}+a_{2}x^{n-2}+\cdots+a_{n}$.

1. *If* *M* *is a mixed tree*, *then* $(-1)^{k}a_{k}=\sum_{M'}(-1)^{r(M')}\prod_{v_{i}\in V(M')}\frac{1}{d_{M_{U}}(v_{i})}$.
2. *If* *M* *is a mixed graph and its underlying graph* $M_{U}$ *is* *r* *regular* ($r\neq0$), *then* $(-1)^{k}a_{k}=\sum_{M'}(-1)^{r(M')+l(M')}2^{s(M')}\frac{1}{r^{k}}$.
3. *If* *M* *is a mixed bipartite graph*, *then all coefficients of* $a_{\mathrm{odd}}$ *are equal to* 0, *and its spectrum is symmetry about* 0.

Note that if *M* is a positive mixed graph, then for every real elementary subgraph $M'$ of *M*, we have

$$\begin{aligned} &(-1)^{r(M')+l(M')}2^{s(M')}\prod_{v_{i}\in V(M')} \frac {1}{d_{M_{U}}(v_{i})} \\ &\quad=(-1)^{r(M')}2^{s(M')}\prod_{v_{i}\in V(M')} \frac {1}{d_{M_{U}}(v_{i})} \\ &\quad=(-1)^{r(M_{U}')}2^{s(M_{U}')}\prod_{v_{i}\in V(M_{U}')} \frac {1}{d_{M_{U}}(v_{i})}. \end{aligned}$$

Then $P_{R_{H}}(M,x)=P_{R_{H}}(M_{U},x)$, that is to say,

### Theorem 2.4

*If* *M* *is a positive mixed graph and* $M_{U}$ *be its underlying graph*, *then* $\operatorname{Sp}_{R_{H}}(M)=\operatorname{Sp}_{R_{H}}(M_{U})$.

## Bounds on the Hermitian-Randić energy of mixed graphs

In this section, we will give some bounds on the Hermitian-Randić energy of mixed graphs. First, we will give some properties of a Hermitian-Randić matrix of mixed graphs.

### Lemma 3.1

*Let* *M* *be a mixed graph of order* $n\geq1$.

1. $\mathcal{E}_{R_{H}}(M)=0$ *if and only if* $M\cong\overline{K}_{n}$.
2. *If* $M=M_{1}\cup M_{2}\cup\cdots\cup M_{p}$, *then* $\mathcal {E}_{R_{H}}(M)=\mathcal{E}_{R_{H}}(M_{1})+\mathcal {E}_{R_{H}}(M_{2})+\cdots+\mathcal{E}_{R_{H}}(M_{p})$.

From Lemma 3.1, we can obtain the following theorem.

### Theorem 3.2

*Let* *M* *be a mixed graph with vertex set* $V(M)=\{v_{1},v_{2},\ldots ,v_{n}\}$, *and* $d_{k}$ *is the degree of* $v_{k}$, $k=1,2,\ldots,n$. *Let* $H(M)$ *and* $R_{H}(M)$ *be the Hermitian*-*adjacency matrix and the Hermitian*-*Randić matrix of* *M*, *respectively*. *If* *M* *has isolated vertices*, *then* $\operatorname{det}H(M)=\operatorname{det}R_{H}(M)=0$. *If* *M* *has no isolated vertices*, *then*

$$\operatorname{det}R_{H}(M)=\frac{1}{d_{1}d_{2}\cdots d_{n}}\operatorname{det}H(M). $$

### Proof

If *M* has *l* isolated vertices, then $M=M'\cup\overline {K}_{l}$, where $M'$ has no isolated vertices. By Lemma 3.1, we have $\operatorname{Sp}_{R_{H}}(M)=\operatorname{Sp}_{R_{H}}(M')\cup\{0, l\text{ times}\}$ and an analogous relation holds for Hermitian-adjacency spectrum of *M*. That is, $H(M)$ and $R_{H}(M)$ have zero eigenvalues, therefore their determinants are equal to zero.

If *M* has no isolated vertices, then $R_{H}(M)=D(M_{U})^{-\frac {1}{2}}H(M)D(M_{U})^{-\frac{1}{2}}$ is applicable, where $D(M_{U})$ is the diagonal matrix of vertex degrees. The matrices $R_{H}(M)$ and $D(M_{U})^{-\frac{1}{2}}R_{H}(M)D(M_{U})^{\frac{1}{2}}$ are similar and thus have equal eigenvalues. We have

$$D(M_{U})^{-\frac{1}{2}}R_{H}(M)D(M_{U})^{\frac{1}{2}}=D(M_{U})^{-1}H(M), $$

therefore,

$$\operatorname{det}R_{H}(M)=\operatorname{det}\bigl[D(M_{U})^{-1}H(M) \bigr]=\operatorname {det}D(M_{U})^{-1}\operatorname{det}H(M). $$

So,

$$\operatorname{det}R_{H}(M)=\frac{1}{d_{1}d_{2}\cdots d_{n}}\operatorname{det}H(M). $$

This completes the proof. □

Similar to Theorem 3.2, we can obtain the following theorem.

### Theorem 3.3

*If* *M* *is a mixed graph with vertex set* $V(M)=\{v_{1},v_{2},\ldots ,v_{n}\}$ *and its underlying graph* $M_{U}$ *is* *r* *regular*, *then* $\mathcal{E}_{R_{H}}(M)=\frac{1}{r}\mathcal{E}_{H}(M)$. *In addition*, *if* $r=0$, *then* $\mathcal{E}_{R_{H}}(M)=0$.

### Proof

If $r=0$, then *M* is the graph that has no edges. Then all the entries of $R_{H}(M)$ are equal to 0, i.e., $R_{H}(M)=\mathbf {0}$. Similarly, $H(M)=\mathbf{0}$. Since all eigenvalues of the zero matrix are equal to 0, hence $\mathcal{E}_{R_{H}}(M)=\mathcal {E}_{H}(M)=0$.

If $r>0$, i.e., *M* is regular of degree $r>0$, then $d_{1}=d_{2}=\cdots =d_{n}=r$, where $d_{k}$ is the degree of $v_{k}$, $k=1,2,\ldots,n$. Hence, $(r_{h})_{sk}=-(r_{h})_{ks}=\frac{\mathbf{i}}{r}$ if $(v_{s},v_{k})$ is an arc of *M*, $(r_{h})_{sk}=(r_{h})_{ks}=\frac {1}{r}$ if $v_{s}v_{k}$ is an undirected edge of *M*, and $(r_{h})_{sk}=0$ otherwise.

This implies that $R_{H}(M)=\frac{1}{r}H(M)$. Therefore, $\mu _{i}=\frac{1}{r}\lambda_{i}$, where $\mu_{i}$ is the eigenvalue of $R_{H}(M)$, and $\lambda_{i}$ is the eigenvalue of $H(M)$ for $i=1,2,\ldots,n$. Then this theorem follows from the definitions of $\mathcal{E}_{R_{H}}(M)$ and $\mathcal{E}_{H}(M)$. □

Similar to the results about the skew Randić energy in [14], we can establish the following lower and upper bounds for the Hermitian-Randić energy. First, we need the following theorem. Here and later, $\mathbf{I}_{n}$ denotes the unit matrix of order *n*.

### Theorem 3.4

*Let* *M* *be a mixed graph of order* *n* *and* $\mu_{1}\geq\mu_{2}\geq\cdots \geq\mu_{n}$ *be the Hermitian*-*Randić spectrum of* $R_{H}(M)$. *Then* $|\mu_{1}|=|\mu_{2}|=\cdots=|\mu_{n}|$ *if and only if there exists a constant* $c=|\mu_{i}|^{2}$ *for all* *i* *such that* $R^{2}_{H}(M)=c \mathbf{I}_{n}$.

### Proof

Let $\mu_{1}\geq\mu_{2}\geq\cdots\geq\mu_{n}$ be the Hermitian-Randić spectrum of $R_{H}(M)$. Then there exists a unitary matrix *U* such that

$$U^{\ast}R_{H}(M)U=U^{\ast}R_{H}(M)^{\ast}U= \operatorname{diag}\{\mu_{1},\ldots ,\mu_{n}\}. $$

So,

$$\begin{aligned} &|\mu_{1}|=|\mu_{2}|=\cdots=|\mu_{n}| \\ &\quad\Leftrightarrow\quad U^{\ast}R_{H}(M)^{\ast}R_{H}(M)U=c \mathbf {I}_{n} \\ &\quad\Leftrightarrow\quad U\bigl(U^{\ast}R_{H}(M)^{\ast}R_{H}(M)U \bigr)U^{\ast}=cUU^{\ast } \\ &\quad\Leftrightarrow\quad R_{H}(M)^{\ast}R_{H}(M)=c \mathbf{I}_{n} \\ &\quad\Leftrightarrow\quad R^{2}_{H}(M)=c \mathbf{I}_{n}, \end{aligned}$$

where *c* is a constant and $c=|\mu_{i}|^{2}$ for all *i*.

This completes the proof. □

### Theorem 3.5

*Let* *M* *be a mixed graph of order* *n* *and* $\mu_{1}\geq\mu_{2}\geq\cdots \geq\mu_{n}$ *be the Hermitian*-*Randić spectrum of* $R_{H}(M)$. *Let* $M_{U}$ *be the underlying graph of* *M*, $p=|\operatorname{det}R_{H}(M)|$. *Then*

$$\sqrt{2R_{-1}(M_{U})+n(n-1)p^{\frac{2}{n}}}\leq\mathcal {E}_{R_{H}}(M)\leq\sqrt{2nR_{-1}(M_{U})} $$

*with equalities holding both in the lower bound and upper bound if and only if there exists a constant* $c=|\mu_{i}|^{2}$ *for all* *i* *such that* $R^{2}_{H}(M)=c \mathbf{I}_{n}$.

### Proof

Let $\{\mu_{1},\mu_{2},\ldots,\mu_{n}\}$ be the Hermitian-Randić spectrum of *M*, where $\mu_{1}\geq\mu_{2}\geq \cdots\geq\mu_{n}$. Since $\sum_{j=1}^{n}\mu_{j}^{2}=\operatorname{tr}(R_{H}^{2}(M))=\sum_{j=1}^{n}\sum_{k=1}^{n}(r_{h})_{jk}(r_{h})_{kj}=\sum_{j=1}^{n}\sum_{k=1}^{n}(r_{h})_{jk}\overline{(r_{h})}_{jk} =\sum_{j=1}^{n}\sum_{k=1}^{n}|(r_{h})_{jk}|^{2}=2R_{-1}(M_{U})$, where $R_{-1}(M_{U})=\sum_{v_{j}v_{k}\in E(M_{U})}\frac{1}{d_{j}d_{k}}$ (unordered).

Applying the Cauchy-Schwarz inequality, we have

$$\mathcal{E}_{R_{H}}(M)=\sum^{n}_{j=1}| \mu_{j}|\leq\sqrt{\sum^{n}_{j=1}| \mu_{j}|^{2}}\cdot\sqrt{n}=\sqrt{2nR_{-1}(M_{U})}. $$

On the other hand,

$$\bigl|\mathcal{E}_{R_{H}}(M)\bigr|^{2}= \Biggl(\sum ^{n}_{j=1}|\mu_{j}| \Biggr)^{2}= \sum^{n}_{j=1}|\mu_{j}|^{2}+ \sum_{1\leq i\neq j\leq n}|\mu _{i}||\mu_{j}|. $$

By using an arithmetic geometric average inequality, we can get that

$$\bigl|\mathcal{E}_{R_{H}}(M)\bigr|^{2}=\sum ^{n}_{j=1}|\mu_{j}|^{2}+\sum _{1\leq i\neq j\leq n}|\mu_{i}||\mu_{j}| \geq2R_{-1}(M_{U})+n(n-1)p^{\frac{2}{n}}. $$

Therefore, we can obtain the lower bound on the Hermitian-Randić energy

$$\mathcal{E}_{R_{H}}(M)\geq\sqrt{2R_{-1}(M_{U})+n(n-1)p^{\frac{2}{n}}}. $$

From the Cauchy-Schwarz inequality and the arithmetic geometric average inequality, we know that the equalities hold both in the lower bound and upper bound if and only if $|\mu_{1}|=|\mu_{2}|=\cdots=|\mu _{n}|$, i.e., there exists a constant $c=|\mu_{i}|^{2}$ for all *i* such that $R^{2}_{H}(M)=c \mathbf{I}_{n}$.

This completes the proof. □

### Corollary 3.6

*Let* *M* *be a mixed graph and its underlying graph* $M_{U}$ *be* *r* (≠0) *regular and* $E(M_{U})=m$. *Let* $\mu_{1}\geq\mu_{2}\geq\cdots\geq\mu _{n}$ *be the Hermitian*-*Randić spectrum of* $R_{H}(M)$. *Then*

$$\sqrt{\frac{n}{r}+n(n-1)p^{\frac{2}{n}}}\leq\mathcal{E}_{R_{H}}(M) \leq \frac{n\sqrt{r}}{r}, $$

*where* $p=|\operatorname{det}R_{H}(M)|$, *with equalities holding both in the lower bound and upper bound if and only if* $\frac{1}{r}=|\mu_{i}|^{2}$ *for all* *i* *such that* $R^{2}_{H}(M)=\frac{1}{r} \mathbf{I}_{n}$.

### Proof

If *M* is a mixed graph and its underlying graph $M_{U}$ is *r* regular, then $R_{-1}(M_{U})=\frac{m}{r^{2}}$ and $2m=nr$. By Theorems 3.4 and 3.5, we can obtain the results. □

### Lemma 3.7

[19]

*Let* *G* *be a graph of order* *n* *with no isolated vertices*. *Then*

$$\frac{n}{2(n-1)}\leq R_{-1}(G)\leq\biggl\lfloor \frac{n}{2} \biggr\rfloor , $$

*with equality in the lower bound if and only if* *G* *is a complete graph*, *and equality in the upper bound if and only if either*

1. *n* *is even and* *G* *is the disjoint union of* $n/2$ *paths of length* 1, *or*
2. *n* *is odd and* *G* *is the disjoint union of* $(n-3)/2$ *paths of length* 1 *and one path of length* 2.

Combining Theorem 3.5 and Lemma 3.7, we can get upper and lower bounds for the Hermitian-Randić energy by replacing $R_{-1}(M_{U})$ with other parameters. We now give bounds of the Hermitian-Randić energy of a mixed graph with respect to its order.

### Theorem 3.8

*Let* *M* *be a mixed graph of order* $n\geq3$ *without isolated vertices and* $M_{U}$ *be its underlying graph*. *Let* $\mu_{1}\geq\mu_{2}\geq\cdots \geq\mu_{n}$ *be the Hermitian*-*Randić spectrum of* $R_{H}(M)$. *Then*

$$\sqrt{\frac{2n}{n-1}}\leq\mathcal{E}_{R_{H}}(M)\leq n. $$

*The equality in the upper bound holds if and only if* *n* *is even and* $M_{U}$ *is the disjoint union of* $n/2$ *paths of length* 1. *The equality in the lower bound holds if and only if* $M_{U}$ *is a complete graph and* $\mu_{1}=-\mu_{n}\neq0$, $\mu_{j}=0$, $j=2,\ldots,n-1$.

### Proof

Let $R_{H}(M)$ be the Hermitian-Randić matrix of *M* and $\mu_{1}\geq\mu_{2}\geq\cdots\geq\mu_{n}$ be the Hermitian-Randić spectrum of $R_{H}(M)$.

For the upper bound, combining Lemma 3.7 and $\mathcal {E}_{R_{H}}(M)\leq\sqrt{2nR_{-1}(M_{U})}$ of Theorem 3.5, we have

$$\mathcal{E}_{R_{H}}(M)\leq\sqrt{2nR_{-1}(M_{U})}\leq \sqrt{2n\biggl\lfloor \frac {n}{2}\biggr\rfloor }\leq n. $$

From Theorem 3.5 and Lemma 3.7, we know that the equality in the upper bound holds if and only if *n* is even, $M_{U}$ is the graph described in Lemma 3.7(1), and $|\mu_{1}|=|\mu_{2}|=\cdots=|\mu_{n}|$, that is, we can obtain the upper bound when *n* is even and $M_{U}$ is the disjoint union of $n/2$ paths of length 1.

For the lower bound, since the sum of the diagonal entries of $R_{H}(M)$ is 0, i.e., $\sum^{n}_{k=1}\mu_{k}=0$, then

$$\begin{aligned} \Biggl(\sum^{n}_{k=1}\mu_{k} \Biggr) \Biggl(\sum^{n}_{l=1} \mu_{l} \Biggr)&=\sum^{n}_{k=1} \mu_{k}^{2}+\sum_{1\leq k\neq l\leq n} \mu_{k}\mu_{l} \\ &=\sum^{n}_{k=1}\mu_{k}^{2}+2 \sum_{k< l}\mu_{k}\mu_{l} \\ &=2R_{-1}(M_{U})+2\sum_{k< l} \mu_{k}\mu_{l} \\ &=0. \end{aligned}$$

Hence, $\sum_{k< l}\mu_{k}\mu_{l}=-R_{-1}(M_{U})$.

From the definition of the Hermitian-Randić energy of a mixed graph, we have

$$\begin{aligned} \mathcal{E}^{2}_{R_{H}}(M)&= \Biggl(\sum ^{n}_{k=1}|\mu_{k}| \Biggr)^{2} \\ &=\sum^{n}_{k=1}\mu_{k}^{2}+ \sum_{1\leq k\neq l\leq n}|\mu_{k}\mu _{l}| \\ &=2R_{-1}(M_{U})+2\sum_{k< l}| \mu_{k}\mu_{l}| \\ &\geq2R_{-1}(M_{U})+2\biggl|\sum_{k< l} \mu_{k}\mu_{l}\biggr| \\ &=4R_{-1}(M_{U}). \end{aligned}$$

Combining this with Lemma 3.7, we have

$$\mathcal{E}_{R_{H}}(M)\geq2\sqrt{R_{-1}(M_{U})}\geq2 \sqrt{\frac {n}{2(n-1)}}=\sqrt{\frac{2n}{n-1}}. $$

So,

$$\sqrt{\frac{2n}{n-1}}\leq\mathcal{E}_{R_{H}}(M)\leq n. $$

From the proof above and Lemma 3.7, we know that the equality in the lower bound holds if and only if $M_{U}$ is a complete graph and $\mu_{k}\mu_{l}\geq0$ or $\mu_{k}\mu _{l}\leq0$ for all $1\leq k< l\leq n$. Note that $\sum_{k=1}^{n}\mu _{k}=0$ and *M* has no isolated vertices, so the former case can not happen. Hence, the equality in the lower bound holds if and only if $M_{U}$ is a complete graph and $\mu_{1}=-\mu_{n}\neq0$, $\mu_{j}=0$, $j=2,\ldots,n-1$.

This completes the proof. □

### Remark 3.9

It should be pointed out that when *M* is a complete mixed graph, its Hermitian-Randić spectrum is not unique. For example, let $M_{U}=K_{3}$, if all edges of $E(M)$ are oriented, then we have $\mu _{1}=-\mu_{3}=\frac{\sqrt{3}}{2}$, $\mu_{2}=0$, then we can obtain the lower bound in Theorem 3.8. If some edges of $E(M)$ are undirected, then we can not obtain the lower bound in Theorem 3.8. For example, if $(r_{h})_{12}=(r_{h})_{32}=\frac{i}{2}$, $(r_{h})_{13}=\frac{1}{2}$, then $\mu_{1}=1$ and $\mu_{2}=\mu_{3}=-\frac {1}{2}$. Hence, the problem of determining all complete mixed graphs for which the lower bound in Theorem 3.8 is attained appears to be somewhat more difficult.

To deduce more bounds on $\mathcal{E}_{R_{H}}(M)$, the following lemma is needed.

### Lemma 3.10

[31]

*Let* $\mathbf{x}, \mathbf{y}\in\mathbb{R}^{n}$ *and let* $A(\mathbf {x})=\frac{1}{n}\sum^{n}_{i=1}x_{i}$, $A(\mathbf{y})=\frac{1}{n}\sum^{n}_{i=1}y_{i}$. *If* $\phi\leq x_{i}\leq\Phi$ *and* $\gamma\leq y_{i}\leq \Gamma$, *then*

$$\Biggl\vert \frac{1}{n}\sum^{n}_{i=1}x_{i}y_{i}- \frac{1}{n^{2}}\sum^{n}_{i=1}x_{i} \sum^{n}_{i=1}y_{i}\Biggr\vert \leq\sqrt{\bigl(\Phi-A(x)\bigr) \bigl(A(x)-\phi \bigr) \bigl(\Gamma-A(y)\bigr) \bigl(A(y)-\gamma\bigr)}. $$

Now we turn to new bounds on $\mathcal{E}_{R_{H}}(M)$.

### Theorem 3.11

*Let* *M* *be a mixed graph of order* *n* *and* $M_{U}$ *be its underlying graph*. *Let* $\mu_{1}\geq\mu_{2}\geq\cdots\geq\mu_{n}$ *be the Hermitian*-*Randić spectrum of* $R_{H}(M)$. *Then*

1 $$ \mathcal{E}_{R_{H}}(M)\geq\frac{2R_{-1}(M_{U})+n\alpha\beta}{\alpha +\beta}, $$

*where* $\alpha=\min_{1\leq i\leq n}\{|\mu_{i}|\}$, $\beta=\max\{\mu _{1},|\mu_{n}|\}$.

### Proof

Note that

2 $$ \begin{aligned}[b] \mathcal{E}^{2}_{R_{H}}(M)&= \Biggl(\sum^{n}_{j=1}|\mu_{j}| \Biggr)^{2} =\sum^{n}_{j=1}|\mu_{j}|^{2}+\sum_{1\leq i\neq j\leq n}|\mu_{i}||\mu _{j}|\\ &=2R_{-1}(M_{U})+\sum_{1\leq i\neq j\leq n}|\mu_{i}||\mu_{j}|. \end{aligned}$$

Let $S=\sum_{1\leq i\neq j\leq n}|\mu_{i}||\mu_{j}|$, $x_{i}=|\mu_{i}|$ and $y_{i}=\mathcal{E}_{R_{H}}(M)-|\mu_{i}|$, $i=1,2,\ldots,n$. Then $S=\sum^{n}_{i=1}x_{i}y_{i}$.

From the definitions of *α* and *β*, we have $\alpha\leq x_{i}\leq\beta$ and $\mathcal{E}_{R_{H}}(M)-\beta\leq y_{i}\leq{\mathcal {E}_{R_{H}}(M)-\alpha}$. In addition, let $A(\mathbf{x})=\frac{1}{n}\sum^{n}_{i=1}x_{i}=\frac{\mathcal{E}_{R_{H}}(M)}{n}$ and $A(\mathbf {y})=\frac{1}{n}\sum^{n}_{i=1}y_{i}=\frac{(n-1)\mathcal {E}_{R_{H}}(M)}{n}$. Hence, by Lemma 3.10, we have

$$\begin{aligned} \biggl\vert \frac{S}{n}-\frac{(n-1)\mathcal{E}^{2}_{R_{H}}(M)}{n^{2}}\biggr\vert \leq{}&\sqrt{ \biggl(\beta-\frac{\mathcal{E}_{R_{H}}(M)}{n} \biggr) \biggl(\frac{\mathcal{E}_{R_{H}}(M)}{n}-\alpha \biggr)} \\ &\cdot\sqrt{ \biggl[\mathcal{E}_{R_{H}}(M)-\alpha-\frac{(n-1)\mathcal {E}_{R_{H}}(M)}{n} \biggr]} \\ &\cdot\sqrt{ \biggl[\frac{(n-1)\mathcal{E}_{R_{H}}(M)}{n}-\bigl(\mathcal {E}_{R_{H}}(M)- \beta\bigr) \biggr]} \\ ={}&\sqrt{ \biggl(\beta-\frac{\mathcal{E}_{R_{H}}(M)}{n} \biggr)^{2} \biggl( \frac{\mathcal{E}_{R_{H}}(M)}{n}-\alpha \biggr)^{2}}. \end{aligned}$$

It follows that

$$S\geq\mathcal{E}^{2}_{R_{H}}(M)+n\alpha\beta-(\alpha+\beta) \mathcal {E}_{R_{H}}(M). $$

This together with (2) implies that

$$\mathcal{E}^{2}_{R_{H}}(M)=2R_{-1}(M_{U})+S \geq2R_{-1}(M_{U})+\mathcal {E}^{2}_{R_{H}}(M)+n \alpha\beta-(\alpha+\beta)\mathcal{E}_{R_{H}}(M). $$

So,

$$\mathcal{E}_{R_{H}}(M)\geq\frac{2R_{-1}(M_{U})+n\alpha\beta}{\alpha +\beta}. $$

This completes the proof. □

Note that the right-hand side of (1) is a non-decreasing function on $\alpha\geq0$. Combining this with Theorem 3.4, we have the following corollary.

### Corollary 3.12

*Let* *M* *be a mixed graph of order* *n* *and* $M_{U}$ *be its underlying graph*. *Let* $\mu_{1}\geq\mu_{2}\geq\cdots\geq\mu_{n}$ *be the Hermitian*-*Randić spectrum of* $R_{H}(M)$. *Then*

$$\mathcal{E}_{R_{H}}(M)\geq\frac{2R_{-1}(M_{U})}{\beta}, $$

*where* $\beta=\max\{\mu_{1},|\mu_{n}|\}$. *The equality holds if and only if* $R^{2}_{H}(M)=c \mathbf{I}_{n}$, *where* *c* *is a constant such that* $|\mu_{i}|^{2}=c$ *for all* *i*.

In particular, if *M* is a connected mixed bipartite graph, then we have the following theorem.

### Theorem 3.13

*Let* *M* *be a connected mixed bipartite graph of order* *n* *and* $M_{U}$ *be its underlying graph*. *Let* $\mu_{1}\geq\mu_{2}\geq\cdots\geq\mu_{n}$ *be the Hermitian*-*Randić spectrum of* $R_{H}(M)$. *Then*

3 $$ \mathcal{E}_{R_{H}}(M)\geq2 \biggl(\frac{R_{-1}(M_{U})+\lfloor\frac {n}{2}\rfloor\alpha\mu_{1}}{\alpha+\mu_{1}} \biggr), $$

*where* $\alpha=\min_{1\leq i\leq\lfloor\frac{n}{2}\rfloor}\{|\mu _{i}|\}$.

### Proof

Note that $M_{U}$ is a bipartite graph. By Corollary 2.3(3), we have $\mu_{i}=-\mu_{n+1-i}$ and $\mu_{i}\geq0$ for $i=1,2,\ldots,\lfloor\frac{n}{2}\rfloor$. Therefore,

4 $$ \begin{aligned}[b]\mathcal{E}^{2}_{R_{H}}(M)&= \Biggl(2\sum^{\lfloor\frac{n}{2}\rfloor }_{i=1}\mu_{i} \Biggr)^{2} =4\Biggl(\sum^{\lfloor\frac{n}{2}\rfloor}_{i=1}\mu_{i}^{2}+\sum_{1\leq i\neq j\leq\lfloor\frac{n}{2}\rfloor}\mu_{i}\mu_{j}\Biggr)\\ &=4R_{-1}(M_{U})+4\sum_{1\leq i\neq j\leq\lfloor\frac{n}{2}\rfloor}\mu _{i}\mu_{j}. \end{aligned} $$

Let $T=\sum_{1\leq i\neq j\leq\lfloor\frac{n}{2}\rfloor}\mu_{i}\mu _{j}$, $x_{i}=\mu_{i}$ and $y_{i}=\frac{\mathcal{E}_{R_{H}}(M)}{2}-\mu _{i}$, $i=1,2,\ldots,\lfloor\frac{n}{2}\rfloor$. Then $T=\sum^{\lfloor\frac{n}{2}\rfloor}_{i=1}x_{i}y_{i}$.

From the definition of *α*, we have $\alpha\leq x_{i}\leq\mu_{1}$ and $\frac{\mathcal{E}_{R_{H}}(M)}{2}-\mu_{1}\leq y_{i}\leq\frac {\mathcal{E}_{R_{H}}(M)}{2}-\alpha$. In addition, let $A(\mathbf {x})=\frac{1}{\lfloor\frac{n}{2}\rfloor}\sum^{\lfloor\frac{n}{2}\rfloor }_{i=1}x_{i}=\frac{\mathcal{E}_{R_{H}}(M)}{2\lfloor\frac{n}{2}\rfloor}$ and $A(\mathbf{y})=\frac{1}{\lfloor\frac{n}{2}\rfloor}\sum^{\lfloor \frac{n}{2}\rfloor}_{i=1}y_{i}=\frac{(\lfloor\frac{n}{2}\rfloor -1)\mathcal{E}_{R_{H}}(M)}{2\lfloor\frac{n}{2}\rfloor}$. Hence, by Lemma 3.10, we have

$$\begin{aligned} \biggl\vert \frac{T}{\lfloor\frac{n}{2}\rfloor}-\frac{(\lfloor\frac {n}{2}\rfloor-1)\mathcal{E}^{2}_{R_{H}}(M)}{4\lfloor\frac{n}{2}\rfloor ^{2}}\biggr\vert &\leq\sqrt{ \biggl(\mu_{1}-\frac{\mathcal {E}_{R_{H}}(M)}{2\lfloor\frac{n}{2}\rfloor} \biggr)^{2} \biggl( \frac {\mathcal{E}_{R_{H}}(M)}{2\lfloor\frac{n}{2}\rfloor}-\alpha \biggr)^{2}}. \end{aligned}$$

It follows that

$$T\geq\frac{\mathcal{E}^{2}_{R_{H}}(M)}{4}+\biggl\lfloor \frac{n}{2}\biggr\rfloor \alpha \mu_{1}-(\alpha+\mu_{1})\frac{\mathcal{E}_{R_{H}}(M)}{2}. $$

This together with (4) implies that

$$\mathcal{E}^{2}_{R_{H}}(M)=4R_{-1}(M_{U})+4T \geq4R_{-1}(M_{U})+\mathcal {E}^{2}_{R_{H}}(M)+4 \biggl\lfloor \frac{n}{2}\biggr\rfloor \alpha\mu_{1}-2(\alpha+\mu _{1})\mathcal{E}_{R_{H}}(M). $$

So,

$$\mathcal{E}_{R_{H}}(M)\geq2 \biggl(\frac{R_{-1}(M_{U})+\lfloor\frac {n}{2}\rfloor\alpha\mu_{1}}{\alpha+\mu_{1}} \biggr). $$

This completes the proof. □

Note that the right-hand side of (3) is a non-decreasing function on $\alpha\geq0$. Combining this with Theorem 3.4, we have the following corollary.

### Corollary 3.14

*Let* *M* *be a connected mixed bipartite graph of order* *n* *and* $M_{U}$ *be its underlying graph*. *Let* $\mu_{1}\geq\mu_{2}\geq\cdots\geq\mu_{n}$ *be the Hermitian*-*Randić spectrum of* $R_{H}(M)$. *Then*

$$\mathcal{E}_{R_{H}}(M)\geq\frac{2R_{-1}(M_{U})}{\mu_{1}}, $$

*the equality holds if and only if* $R^{2}_{H}(M)=c \mathbf {I}_{n}$, *where* *c* *is a constant such that* $|\mu_{i}|^{2}=c$ *for all i*.

## Hermitian-Randić energy of trees

In [21], the authors proved that the skew energy of a directed tree is independent of its orientation. In [14], the authors showed that the skew Randić energy of a directed tree has the same result. In this section, we will show that the Hermitian-Randić energy also has the same result. In the beginning of this section, we first characterize the mixed graphs with cut-edge.

### Theorem 4.1

*Let* *M* *be a mixed graph of order* *n*, *and* $e=uv$ *is an edge of* *M*. *If* *uv* *is a cut*-*edge of* $M_{U}$, *where* $M_{U}$ *is the underlying graph of* *M*, *then the spectrum and energy of* $R_{H}(M)$ *are unchanged when the edge* *uv* *is replaced with a single arc* *uv* *or* *vu* *and vice versa*.

### Proof

Let $e=uv$ be a cut-edge of $M_{U}$. Suppose that $M_{1}$ is the graph obtained from *M* by replacing the edge *uv* with the arc *uv* or *vu*. Let $M'$ and $M_{1}'$ be real elementary subgraphs of order *k* of *M* and $M_{1}$, respectively. If $M'$ does not contain the cut-edge *uv*, then $M'$ is also a real elementary subgraph of $M_{1}$, that is, $M'=M_{1}'$. By Theorem 2.2, we have

5 $$ \begin{aligned}[b]&(-1)^{r(M')+l(M')}2^{s(M')}\prod_{v_{i}\in V(M')}\frac {1}{d_{M_{U}}(v_{i})}\\ &\quad=(-1)^{r(M_{1}')+l(M_{1}')}2^{s(M_{1}')}\prod_{v_{i}\in V(M_{1}')}\frac {1}{d_{M_{U}}(v_{i})}. \end{aligned} $$

If $M'$ contains the cut-edge *uv*, then there is a real elementary subgraph $M_{1}'$ of $M_{1}$ only different from $M'$ on *uv*. Since *uv* is a cut-edge of $M_{U}$, *uv* is not contained in any cycles of $M_{U}$. Hence, by Theorem 2.2, we have

6 $$ \begin{aligned}[b]&(-1)^{r(M')+l(M')}2^{s(M')}\prod_{v_{i}\in V(M')}\frac {1}{d_{M_{U}}(v_{i})}\\ &\quad=(-1)^{r(M_{1}')+l(M_{1}')}2^{s(M_{1}')}\prod_{v_{i}\in V(M_{1}')}\frac {1}{d_{M_{U}}(v_{i})}. \end{aligned} $$

Combining (5) and (6), we have $a_{k}(M)-a_{k}(M_{1})=0$ for any integer *k*.

Thus $\operatorname{Sp}_{R_{H}}(M)=\operatorname{Sp}_{R_{H}}(M_{1})$. Moreover, $\mathcal {E}_{{R_{H}}}(M)=\mathcal{E}_{{R_{H}}}(M_{1})$.

Similarly, we can prove that $\operatorname{Sp}_{R_{H}}(M)=\operatorname{Sp}_{R_{H}}(M_{2})$ and $\mathcal{E}_{{R_{H}}}(M)=\mathcal{E}_{{R_{H}}}(M_{2})$, where $M_{2}$ is the mixed graph obtained from *M* by replacing the arc *uv* or *vu* with the edge *uv*. □

Thus, the Hermitian-Randić spectrum and the Hermitian-Randić energy are invariants when reversing the cut-arc’s orientation or unorienting it or orienting an undirected cut-edge. By applying Theorem 4.1, we can obtain the following corollaries.

### Corollary 4.2

*Let* *T* *be a mixed tree of order* *n* *and* $T'$ *be the mixed tree obtained from* *T* *by reversing the orientations of all the arcs incident with a particular vertex of* *T*. *Then* $\mathcal{E}_{R_{H}}(T)=\mathcal{E}_{R_{H}}(T')$.

### Corollary 4.3

*Let* *T* *be a mixed tree and* $T_{U}$ *be its underlying graph*. *Then*

1. *The Hermitian*-*Randić energy of* *T* *is independent of its orientation of the arc set*.
2. *The Hermitian*-*Randić energy of* *T* *is the same as the Randić energy of* $T_{U}$.

## Conclusions

In this paper, we define the Hermitian-Randić matrix of a mixed graph *M* and give the definitions of Hermitian-Randić characteristic polynomial and Hermitian-Randić energy of a mixed graph *M*. We give the bounds on the Hermitian-Randić energy of a mixed graph *M* with respect to its order, the Hermitian-Randić spectrum and a general Randić index (with $\alpha=-1$). We also obtain that the Hermitian-Randić energy of a mixed tree is the same as the Randić energy of its underlying graph.

Our future work will focus more on the characterizations of the Hermitian-Randić matrix of mixed graphs, such as the Hermitian-Randić spectrum of a complete mixed graph, more bounds on the Hermitian-Randić energy of mixed graphs with other parameters and mixed graphs that share the same Hermitian-Randić spectra with their underlying graphs.
